# Type 1 Hyperphosphatemic Familial Tumoral Calcinosis Associated With a Homozygous Variant Mutation in the GALNT3 Gene

**DOI:** 10.7759/cureus.71390

**Published:** 2024-10-13

**Authors:** Somayah A Alghubishi, Eman J Ghazwani, Sami E Abdelmogeit, Khalid Alzubair

**Affiliations:** 1 Pediatrics, Armed Forces Hospital Southern Region, Khamis Mushait, SAU; 2 Pediatric Infectious Diseases, Armed Forces Hospital Southern Region, Khamis Mushait, SAU

**Keywords:** calcinosis, galnt3 mutation, genetic mutation, hyperphosphatemia, hyperphosphatemic familial tumoral calcinosis, inherited calcinosis, multifocal osteomyelitis, osteomyelitis, rare genetic mutation

## Abstract

Hyperphosphatemic familial tumoral calcinosis (HFTC) is a rare genetic disorder characterized by abnormal phosphate metabolism leading to hyperphosphatemia and calcific deposits in soft tissues. Chronic recurrent multifocal osteomyelitis (CRMO) can be challenging to diagnose and manage, especially in the context of underlying genetic conditions. This case report presents a case of a 12-year-old girl with a complex presentation involving osteomyelitis and a rare genetic disorder. This 12-year-old girl was referred by the orthopedic team for evaluation of right tibial osteomyelitis based on MRI findings. She had experienced painful swelling, redness, and increased warmth in her right thigh a month prior, which improved with a nonsteroidal anti-inflammatory drug (NSAID) (Ibuprofen) alone. She became asymptomatic without the need for antibiotics and did not have a fever or respiratory symptoms during this episode. The physical examination revealed an alert and oriented patient with no dysmorphic features. Notable findings included a scar on the right thigh from the previous surgery and multiple small lesions on the pubic area. Her height and weight were appropriate for her age. MRI suggested right tibial osteomyelitis. Laboratory studies showed hyperphosphatemia and whole exome sequencing (WES) identified HFTC type 1. The patient’s presentation of right tibial osteomyelitis, initially thought to be CRMO, was ultimately explained by the diagnosis of HFTC type 1, as revealed by WES. This genetic condition, associated with hyperphosphatemia and calcific deposits, accounts for her recurrent osteomyelitis, systemic symptoms, and previous bone tumor history. Management should focus on addressing phosphate imbalances and monitoring for related complications, with input from a geneticist and a specialist in metabolic bone disorders to guide comprehensive care.

## Introduction

Hyperphosphatemic familial tumoral calcinosis (HFTC) was first identified as a distinct condition from other tumoral calcinosis entities in 1996 [1]. There is currently no known prevalence, but it is more frequently observed in Africa and the Middle East [2]. Calcifications usually form during childhood [3]. Regarding pathogenesis, the kidneys are unable to excrete phosphorus due to a loss of function of fibroblast growth factor-23 (FGF-23), which normally prevents its reabsorption. Subsequently, the increase in serum phosphorus levels leads to the development of calcium-phosphate complexes that accumulate in soft tissues, particularly around the joints [4]. Three genes, Klotho (KL), UDP-N-acetyl-alpha-D-galactosamine polypeptide N-acetylgalactosamine transferase 3 (GALNT3), and GF23, have been found to be responsible for causing HFTC. FGF23 encodes the phosphaturic hormone FGF-23 [5], GALNT3 encodes UDP-N-acetyl-alpha-D-galactosamine polypeptide N-acetylgalactosamine transferase 3, and KL encodes alpha Klotho [6]. There are only three reports in the literature that link HFTC types 2-4 with chronic recurrent multifocal osteomyelitis (CRMO), which is an autoinflammatory disorder characterized by bone pain and fever [7]. Osteomyelitis is the most common type of bone infection [8]. Every year, a small number of children, mostly under the age of five, are affected by this infection [8]. It typically occurs in the metaphysis of long bones, such as the humerus, tibia, and femur [9]. An appropriate approach for patients who are resistant to parenteral antibiotics is surgery followed by treatment with antibiotics [10]. Typically, antibiotic therapy lasts for four to eight weeks [11]. However, there have been cases where positive results were achieved in simpler situations with an average treatment duration of just 23 days [11]. The course of the disease is unpredictable, with periods of exacerbations and spontaneous remissions [11]. This case report presents a 12-year-old female with a complex presentation involving osteomyelitis and a rare genetic disorder called HFTC.

## Case presentation

A 12-year-old female patient was referred by the orthopedic team for evaluation of right tibial osteomyelitis, as indicated by MRI findings. She presented with a history of painful swelling, redness, and increased warmth in her right thigh that developed one month prior. These symptoms were managed successfully with a nonsteroidal anti-inflammatory drug (NSAID) (ibuprofen) alone, and she became asymptomatic without requiring antibiotics. Notably, she did not experience fever or respiratory symptoms during this episode.

**Figure 1:**
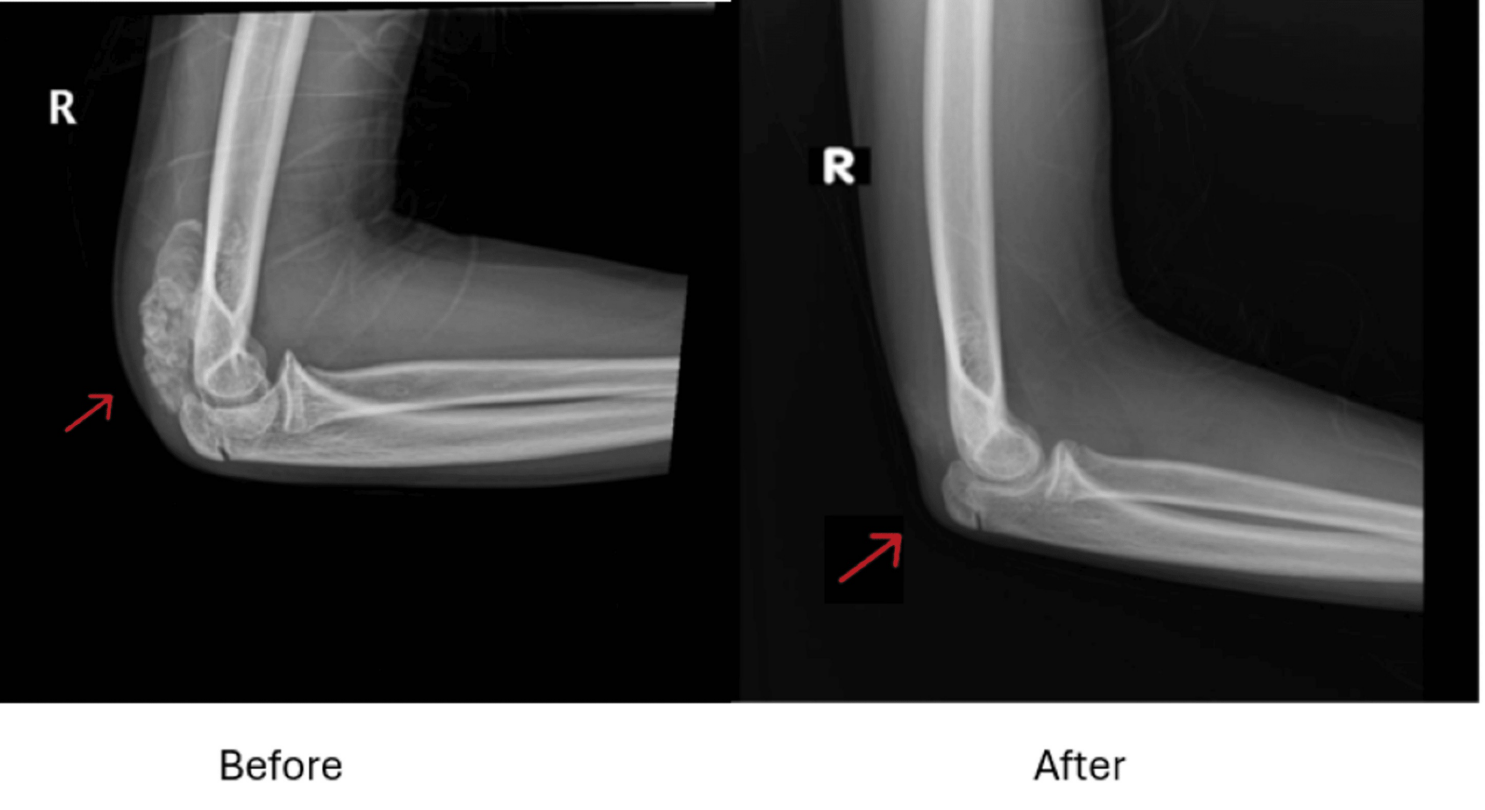
X-ray of Right Elbow Before and After Treatment for Hyperphosphatemic Familial Tumoral Calcinosis The "Before" image shows significant calcific deposits near the olecranon process of the right elbow (red arrow), characteristic of tumoral calcinosis. The "After" image demonstrates a marked reduction in the calcific mass following treatment, highlighting the efficacy of the intervention in reducing phosphate-related calcific deposits.

Her aunt had a benign tumor and underwent resection at the age of 30, and she has been doing well since then. The patient was admitted again with soft tissue tumors in her right elbow and underwent resection (Figure 1). The radiographic comparison of the patient's right elbow before and after treatment (Figure 1) demonstrates a significant reduction in calcific deposits, consistent with the therapeutic management of HFTC, highlighting the importance of addressing phosphate dysregulation to mitigate soft tissue calcification.

On the basis of these test results, the following recommendations were made: Targeted testing for the parents was advised to confirm their carrier status and the homozygosity of the identified variant. Testing for affected family members, if any, was recommended to identify individuals who may be carriers or affected by the same genetic condition.

Additionally, comprehensive genetic counseling was recommended. This included discussions on reproductive options, such as prenatal and preimplantation genetic diagnosis, to provide guidance on future family planning. Initially, the clinical impression was CRMO due to the patient's history of similar complaints and multisystem involvement. However, the genetic findings pointed to HFTC type 1, which better explained the patient’s recurrent osteomyelitis and systemic symptoms. The patient's past medical history includes the resection of a right thigh bone tumor 10 months ago. Histopathological examination of the tumor revealed micro-calcification and a secondary multinucleated giant cell reaction. Additionally, she had a supra-pubic skin lesion that was biopsied and diagnosed as pseudoxanthoma elasticum. Family history is notable for first-degree consanguinity, with the mother having a known case of hypothyroidism that is currently managed with medication.

**Figure 2:**
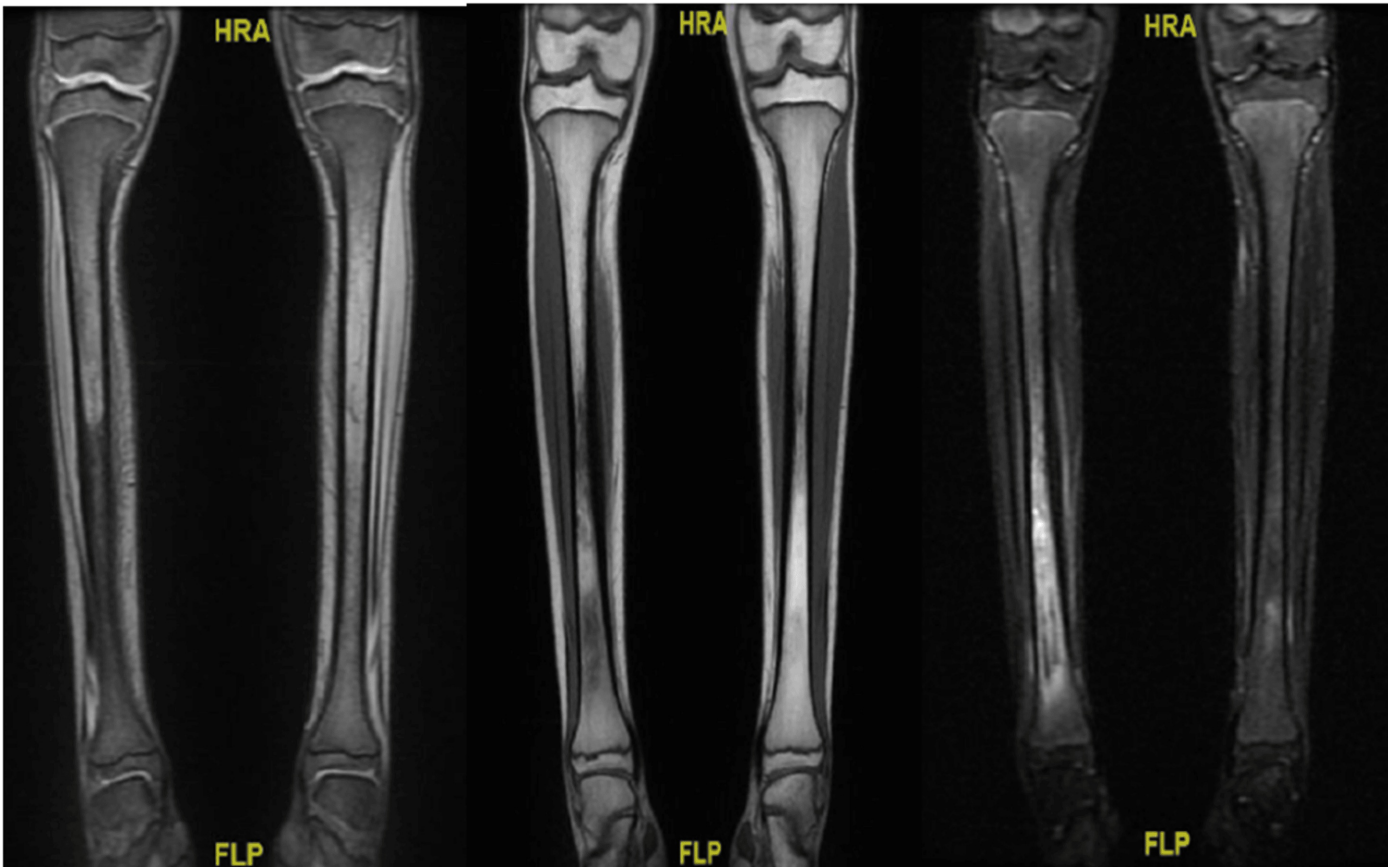
MRI of the Right Tibial Shaft Showing Diffuse Altered Bone Marrow Signal With Soft Tissue Changes MRI of the right tibial shaft shows a diffuse altered bone marrow signal, characterized by heterogeneous low T1 and bright T2/short tau inversion recovery (STIR) signals with enhancement. Cortical thickening and mild edematous changes in the surrounding soft tissue are also noted. HRA: high-resolution anatomical; FLP: finger-like projection

On review of systems, the patient had no history of trauma, raw milk ingestion, or contact with sick individuals. Physical examination showed that the patient was alert and oriented with no dysmorphic features. Local findings included a scar on the right thigh from the previous surgery and multiple small lesions on the pubic area. Her height and weight were within the expected range for her age. Diagnostic evaluations included an MRI, which suggested right tibial osteomyelitis (Figure 2).

Laboratory studies revealed hyperphosphatemia and whole exome sequencing (WES) identified a homozygous pathogenic variant in the GALNT3 gene, confirming the diagnosis of autosomal recessive HFTC type 1. No additional clinically relevant variants related to the described phenotype were detected.

## Discussion

Hyperphosphatemic tumoral calcinosis (HTC) is a rare hereditary disorder characterized by the abnormal deposition of calcium phosphate in soft tissues, typically around joints [12]. This condition results from mutations in genes involved in phosphate regulation, such as GALNT3, FGF23, and KL [13,14]. The pathogenic mechanism involves disrupted phosphate homeostasis, leading to hyperphosphatemia and ectopic calcifications. Clinically, HTC presents with painful, progressive masses, most commonly affecting the hips, shoulders, and elbows [15-18]. Early diagnosis and genetic evaluation are essential for appropriate management, which often focuses on lowering serum phosphate levels and preventing further calcifications.

Mutations in GALNT3 have also been linked to hyperphosphatemia hyperostosis syndrome (HHS), a condition characterized by periodic leg swelling, leg pain, sclerosis, periosteal reaction, and cortical hyperostosis. This condition shares similarities with CRMO [19]. In our case, WES testing results revealed mutations in GALNT3. Previous studies have described the coexistence of HHS and HFTC conditions in the same patients, suggesting that these manifestations may be different presentations of the same underlying condition [20].

It is worth speculating whether the clinical and radiological findings of these children with HHS could indicate a possible connection or range of CRMO. The mutation c.1524+1G>A in GALNT3 has been previously reported to cause HFTC in a Druze family with tumoral calcinosis and in Arab Muslim families with HHS and HFTC [16]. Discovering the c.1524+1G>A mutation in a patient from Jordan, as well as in other Druze families and Arab Muslims, indicates a possible founder effect and implies that this mutation originated from a common source in the Middle East. In addition, it is worth noting that not all families in the study had a diagnosis of CRMO, indicating that there may be genetic factors at play that influence the presentation of the disease.

There is limited information on the most effective treatment for HFTC, including options like a low-phosphate diet or the use of phosphate binders like sevelamer and calcium carbonate [16]. Additional treatments such as acetazolamide and bisphosphonates have varying degrees of success in managing disease activity. According to a recent case report, it was found that acetazolamide did not have an effect on phosphate levels. However, it was observed that it increased the solubility of calcium phosphate complexes by inducing metabolic acidosis [21].

## Conclusions

The patient’s presentation of right tibial osteomyelitis, initially thought to be CRMO, was ultimately explained by the diagnosis of HFTC type 1, as revealed by WES. This genetic condition, associated with hyperphosphatemia and calcific deposits, accounts for her recurrent osteomyelitis, systemic symptoms, and previous bone tumor history. Management should focus on addressing phosphate imbalances and monitoring for related complications, with input from a geneticist and a specialist in metabolic bone disorders to guide comprehensive care.
